# Gasdermin D could be lost in the brain parenchyma infarct core and a pyroptosis-autophagy inhibition effect of Jie-Du-Huo-Xue decoction after stroke

**DOI:** 10.3389/fphar.2024.1449452

**Published:** 2024-07-29

**Authors:** Chang Zhou, Shi-wei Qiu, Feng-ming Wang, Yu-chen Liu, Wei Hu, Mei-lan Yang, Wang-hua Liu, Hua Li

**Affiliations:** ^1^ Hunan University of Chinese Medicine, Changsha, Hunan, China; ^2^ Provincial Key Laboratory of TCM Diagnostics, Hunan University of Chinese Medicine, Changsha, Hunan, China; ^3^ Key Laboratory of Hunan Province for Integrated Traditional Chinese and Western Medicine on Prevention and Treatment of Cardio-Cerebral Diseases, Changsha, Hunan, China; ^4^ Key Laboratory of TCM Heart and Lung Syndrome Differentiation & Medicated Diet and Dietotherapy, University of Chinese Medicine, Changsha, Hunan, China; ^5^ Hunan Engineering Technology Research Center for Medicinal and Functional Food, Hunan University of Chinese Medicine, Changsha, Hunan, China

**Keywords:** stroke, cerebral ischemia-reperfusion injury, pyroptosis, autophagy, NLRP3

## Abstract

**Background:**

The Chinese ethnic medicine Jie-Du-Huo-Xue Decoction (JDHXD) is used to alleviate neuroinflammation in cerebral ischemia (CI). Our previous studies have confirmed that JDHXD can inhibit microglial pyroptosis in CI. However, the pharmacological mechanism of JDHXD in alleviating neuroinflammation and pyroptosis needs to be further elucidated. New research points out that there is an interaction between autophagy and inflammasome NLRP3, and autophagy can help clear NLRP3. The NLRP3 is a key initiator of pyroptosis and autophagy. The effect of JDHXD promoting autophagy to clear NLRP3 to inhibit pyroptosis on cerebral ischemia-reperfusion inflammatory injury is currently unknown. We speculate that JDHXD can inhibit pyroptosis in CI by promoting autophagy to clear NLRP3.

**Methods:**

Chemical characterization of JDHXD was performed using LC-MS. Model of middle cerebral artery occlusion/reperfusion (MCAO/R) was established in SD rats. Neurological deficits, neuron damage, and cerebral infarct volume were evaluated. Western Blot and immunofluorescence were used to detect neuronal pyroptosis and autophagy.

**Results:**

30 possible substance metabolites in JDHXD medicated serum were analyzed by LC-MS (Composite Score > 0.98). Furthermore, JDHXD protects rat neurological function and cerebral infarct size after CI. JDHXD inhibited the expression of pyroptosis and autophagy after CI. Our western blot and immunofluorescence results showed that JDHXD treatment can reduce the expression of autophagy-related factors ULK1, beclin1, and LC3-Ⅱ. The expression of NLRP3 protein was lower in the JDHXD group than in the I/R group. Compared with the I/R group, the expressions of pyroptosis-related factors caspase-1 P 10, GSDMD-NT, IL-18, and IL-1β decreased in the JDHXD group. Furthermore, we observed an unexpected result: immunofluorescence demonstrated that Gasdermin D (GSDMD) was significantly absent in the infarct core, and highly expressed in the peri-infarct and contralateral cerebral hemispheres. This finding challenges the prevailing view that GSDMD is elevated in the ischemic cerebral hemisphere.

**Conclusion:**

JDHXD inhibited pyroptosis and autophagy after MCAO/R. JDHXD suppressed pyroptosis and autophagy by inhibiting NLRP3, thereby alleviating CI. In addition, we present a different observation from previous studies that the expression of GSDMD in the infarct core was lower than that in the peri-infarct and contralateral non-ischemic hemispheres on day 3 of CI.

## Introduction

“To die or not to die, that is the question,” as Shakespeare said in *Hamlet*. How neurons die is also a question in stroke research. Stroke stands as the foremost cause of mortality and acquired physical impairment worldwide. The range of available treatments remains limited to recanalization therapies, which include systemic thrombolysis and interventional thrombectomy (Haupt et al., 2023). Recanalizing techniques, however, are restricted to short time frames and a certain imaging profile of stroke. Therefore, the development of neuroprotectants remains important. Multitude potential targets for neuroprotection in the cascade of ischemic stroke have been identified, encompassing neuroinflammation, blood-brain barrier (BBB) permeability, and diverse cell death (Huang et al., 2020). Management remains challenging because the pathophysiology of CI is complex and involves multiple cascades. Until now, numerous potential neuroprotective medications have exhibited encouraging preclinical data. Regrettably, none have successfully transitioned into routine clinical practice (Haupt et al., 2023). However, modulating programmed cell death to rescue penumbra neurons remains important in stroke research. In recent years, the newly discovered crosstalk between cell death has stimulated our strong research interest.

Jie-Du-Huo-Xue decoction (JDHXD), was created by Qing-ren Wang, a renowned physician of the Qing Dynasty and the author of *Correction of Errors in Medical Classics*. JDHXD is a traditional Chinese medicine formula utilized for reducing inflammation in CI, comprising 10 botanical drugs (Table 1), and it is still a classic prescription in botanical medicine today. The above plants are not unique to the Chinese medical system and are used as ethnic medicines in Russia, European countries, and Japan (Fiore et al., 2005; Shikov et al., 2014; Nogami-Hara et al., 2018; Shikov et al., 2021). Our previous study confirmed that JDHXD has a neuroprotective effect on CIRI by inhibiting microglial pyroptosis and promoting microglial M1-M2 phenotype transformation (Zhou et al., 2023). Pyroptosis is triggered by a wide range of infectious and non-infectious stimuli (Vercammen et al., 1998; Bergsbaken et al., 2009; Gou et al., 2021). Upon the onset of CI, the body initiates the pattern signal associated with damage-associated molecular patterns (DAMPs) (Fricker et al., 2018). Subsequently, the intracellular promoter protein nucleotide-binding oligomerization domain-like receptor pyrin domain containing 3 (NLRP3) can recruit pro-caspase-1 (Sun et al., 2020; Yang et al., 2022). When caspase-1 is formed, the active caspase-1 cleaves the protein GSDMD to activate it. Activated GSDMD accumulates in the cell membrane and forms pores (Burdette et al., 2021). Meanwhile, caspase-1 can additionally trigger the maturation of pro-IL-1β and pro-IL-18. Upon maturation, IL-1β and IL-18 are released into the extracellular space, consequently attracting additional inflammasomes (Luan et al., 2020), following the aggregation of inflammasomes, the inflammatory response escalates, exacerbating tissue damage further. For example, IL-1β can damage the BBB by inducing microglia-mediated inflammatory responses (Niu et al., 2023).

**TABLE 1:** Botanicals of Jie-Du-Huo-Xue decoction.

Standard name	English name	Chinese name	Part used	Grammage (g, %)	Cat#
*Pueraria edulis* Pamp [Fabaceae]	*Pueraria lobata root*	*Gegen*	Root	15 g (13.76%)	CK22082901
*Paeonia veitchii* Lynch [Paeoniaceae]	*Red peony root*	*Chishao*	Root	15 g (13.76%)	SL22081501
*Prunus persica* (L.) Batsch [Rosaceae]	*Peach seed*	*Taoren*	Seeds	12 g (11.01%)	2022070201
*Anethum graveolens* L. [Apiaceae]	*Chinese angelica*	*Danggui*	Root	15 g (13.76%)	TH22091903
*Rehmannia glutinosa (Gaertn.)* DC. [Orobanchaceae]	*Rehmannia root*	*Dihuang*	Root	15 g (13.76%)	2022090401
*Carthamus tinctorius* L. [Asteraceae]	*Safflower*	*Honghua*	Flower	10 g (9.17%)	2022080504
*Forsythia suspensa (Thunb.)* Vahl [Oleaceae]	*Forsythia suspensa*	*Lianqiao*	Gains	15 g (13.76%)	RS22080501
*Bupleurum falcatum* L. [Apiaceae]	*Chinese thorowax root*	*Chaihu*	Root	10 g (9.17%)	CK22091401
*Citrus medica* L. [Rutaceae]	*Immature trifoliate-orange fruit*	*Zhiqiao*	Gains	6 g (5.5%)	XZ22083002
*Glycyrrhiza glabra* L. [Fabaceae]	*Licorice*	*Gancao*	Root	6 g (5.5%)	NG22090503

Autophagy, an intracellular degradation process essential for cellular homeostasis, occurs extensively in ischemic stroke through autolysosome degradation of damaged or aged organelles and proteins (Shen et al., 2021; Shi et al., 2021). The regulation of autophagy involves damage sensors, often converging on the mechanistic target of rapamycin complex 1 (MTORC1), a primary inhibitor of autophagy (Plaza-Zabala et al., 2017). During classical autophagy, MTORC1 inhibition promotes activation of the autophagy pre-initiation complex comprising the unc-51-like autophagy activating kinase 1 (ULK-1). phosphate, essential for forming the pre-autophagosomal membrane or phagophore (Zhai et al., 2023). The synchronized action of autophagy-related proteins (ATGs) regulates the elongation of this structure, resulting in the development of the autophagosome. Consequently, ATGs lipidate microtubule-associated light chain 3 (LC3), leading to its accumulation in autophagosomal membranes, where it aids in cargo engulfment by binding to autophagic substrates (Hou et al., 2019; Zhang et al., 2020).

Interestingly, the NLRP3-mediated canonical pyroptosis pathway is influenced by autophagy, because autophagy can remove NLRP3, thereby inhibiting pyroptosis (Biasizzo and Kopitar-Jerala, 2020; Liu et al., 2021; Ji et al., 2022). The inhibition of pyroptosis by autophagy is primarily achieved through three mechanisms. Firstly, autophagy reduces apoptosis-associated speck-like protein containing a card (ASC). Secondly, it phosphorylates NLRP3. Thirdly, autophagy removes reactive oxygen species (ROS) (Zhao S. et al., 2021). NLRP3, ASC, and ROS, which are inhibited by autophagy, are all key initiators of pyroptosis. The accumulation of ROS is one of the most important pathophysiological processes of CIRI, and ROS can promote the palmitoylation of GSDMD and aggravate pyroptosis (Du et al., 2024). The inflammasome complex composed of NLRP3 and ASC is the initiator of the classic NLRP3/caspase-1/GSDMD pyroptosis pathway. Research on the interaction between pyroptosis and autophagy based on the inflammasome NLRP3 has been carried out in cardiovascular and metabolic diseases (Liu et al., 2019; Hu et al., 2022).

The above inspired us to study the mechanism of JDHXD on CIRI further. Based on our previous confirmation that JDHXD can inhibit pyroptosis in rats on the third day of CI, we hypothesized that JDHXD alleviates CI inflammatory damage by promoting autophagy to inhibit NLRP3-mediated classical pyroptosis. Therefore, the present study was conducted to confirm whether JDHXD alleviates inflammatory damage following CI by promoting autophagy to inhibit NLRP3-mediated pyroptosis.

## Materials and methods

### Composition and quality control for JDHXD

Our earlier research found that JDHXD has a significant applicability value in ischemic stroke. Our previous studies have described the specific composition, preparation method, and HPLC spectrum of JDHXD (Zhou et al., 2023). All botanicals (listed in Table 1) were obtained from the First Affiliated Hospital of Hunan University of Chinese Medicine pharmacies. The purchased drugs were identified in source, properties, physicochemical properties, authenticity, purity, and quality. Samples of the drugs were stored in the Key Laboratory of Chinese Medicine Diagnostics of Hunan Province. The full standard names have been checked through the following website, http://mpns.kew.org/mpns-portal/. The preparation method of JDHXD decoction is as follows: mix the required ten kinds of botanicals, add an appropriate amount of water, soak for 2 h, and decoct in water. Use 6 times the water for the first time and decoct for 1.5 h; use 3 times the water for the second time and decoct for 1.5 h. Mix the two decoctions and concentrate them to a crude drug concentration of 1 g/mL.

### Animals

A total of 96 male SD rats were used in this experiment. After excluding 24 rats that failed to establish the model, the remaining 72 rats were included in subsequent experiments. The reason for using male rats in this study is that estrogen can protect against CI (Niu et al., 2023). Efforts were also taken to reduce animal suffering during the trial. The Hunan University of Chinese Medicine Laboratory Animal Research Center provided specific pathogen-free male Sprague-Dawley rats weighing 250–280 g (No: 430727221102014621 and 430727221102520668). The rats were maintained in a standard SPF barrier environment at 23°C with a light-dark cycle of 12:12 h. Three rats were housed in a single cage, with the animals permitted to consume food and water at their discretion. Before drug administration or surgery, rats were fed adaptively for 3 days in advance, and feeding was stopped 1 day before surgery, but drinking water was not restricted. If the neurological function injury score of a rat exceeds three points following modeling, or if it is predicted that the animal will not survive until the conclusion of the experiment, such rats will be euthanized by an overdose of anesthesia. Due to the limitations of the MCAO/R model and the rats will be killed for sampling 3 days after modeling, no special postoperative treatment will be performed on rats with CI.

### Drug administration and experimental design

Briefly, the equivalent dose for rats is calculated as 10.71 g/kg/day based on the ratio of body surface area between humans and rats. Subsequently, we established the low, medium, and high dosage JDHXD for the current experiment as 5.36, 10.71, and 21.42 g/kg/day respectively. The volume for gavage administration was calculated at 2 mL per 200 g body weight. Preparation of low, medium, and high concentrations of JDHXD was done according to the rat dosages. Edaravone 3.2 mg/kg/day was administered intraperitoneally to rats in the positive drug group.

In the first stage of this study, 36 rats were randomly divided into sham operation group (Sham), ischemia and reperfusion group (I/R), JDHXD low-dosage group (JDHXD-L), JDHXD medium-dosage group (JDHXD-M), JDHXD high-dosage group (JDHXD-H) and Edaravone group by random number table method. The number of rats required in each group was calculated based on three rats for each detection method. The rats in the JDHXD-treated groups were orally administered the corresponding dosage of a concentrated solution of JDHXD, and the other rats were given the same volume of distilled water. The administration of the drug occurred twice daily at 9:00 and 16:00 for 3 days before the surgery and continued for 3 days after the surgery until the animals were sacrificed. Following the assessment of neurological function and the cerebral infarct area, the optimal dosage of JDHXD (JDHXD-M) was chosen for subsequent experiments.

**FIGURE 1:**
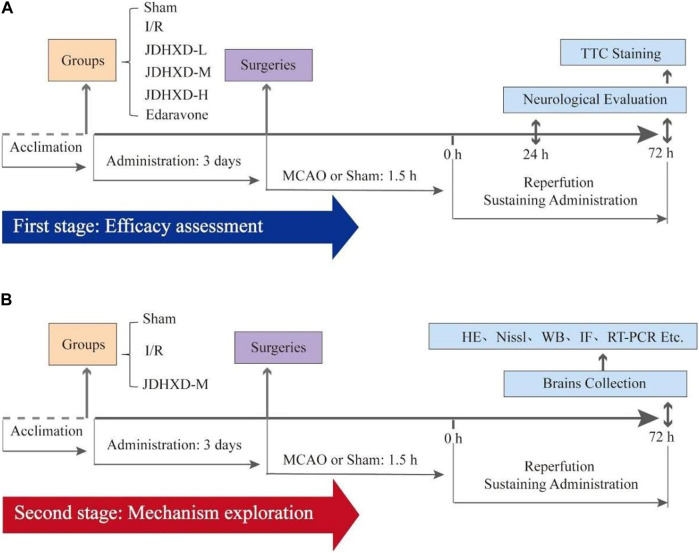
This schematic diagram of the present study. **(A)** The first stage evaluates Jie-Du-Huo-Xue decoction’s (JDHXD) efficiency against ischemia-reperfusion injury. **(B)** The second phase of this study aimed to investigate the interactive effects of JDHXD on pyroptosis and autophagy.

During the second stage of this trial, 36 rats were randomly allocated to one of three groups: the sham group (Sham), the ischemia and reperfusion group (I/R), and the JDHXD medium-dosage group (JDHXD-M). For the JDHXD-M animal, drug administration took place as previously reported 3 days before the surgery and the last 3 days post-reperfusion. The rats were beheaded 72 h after the surgeries. Figure 1 depicts the theoretical diagram for the research.

### Cerebral ischemia-reperfusion injury models

**FIGURE 2:**
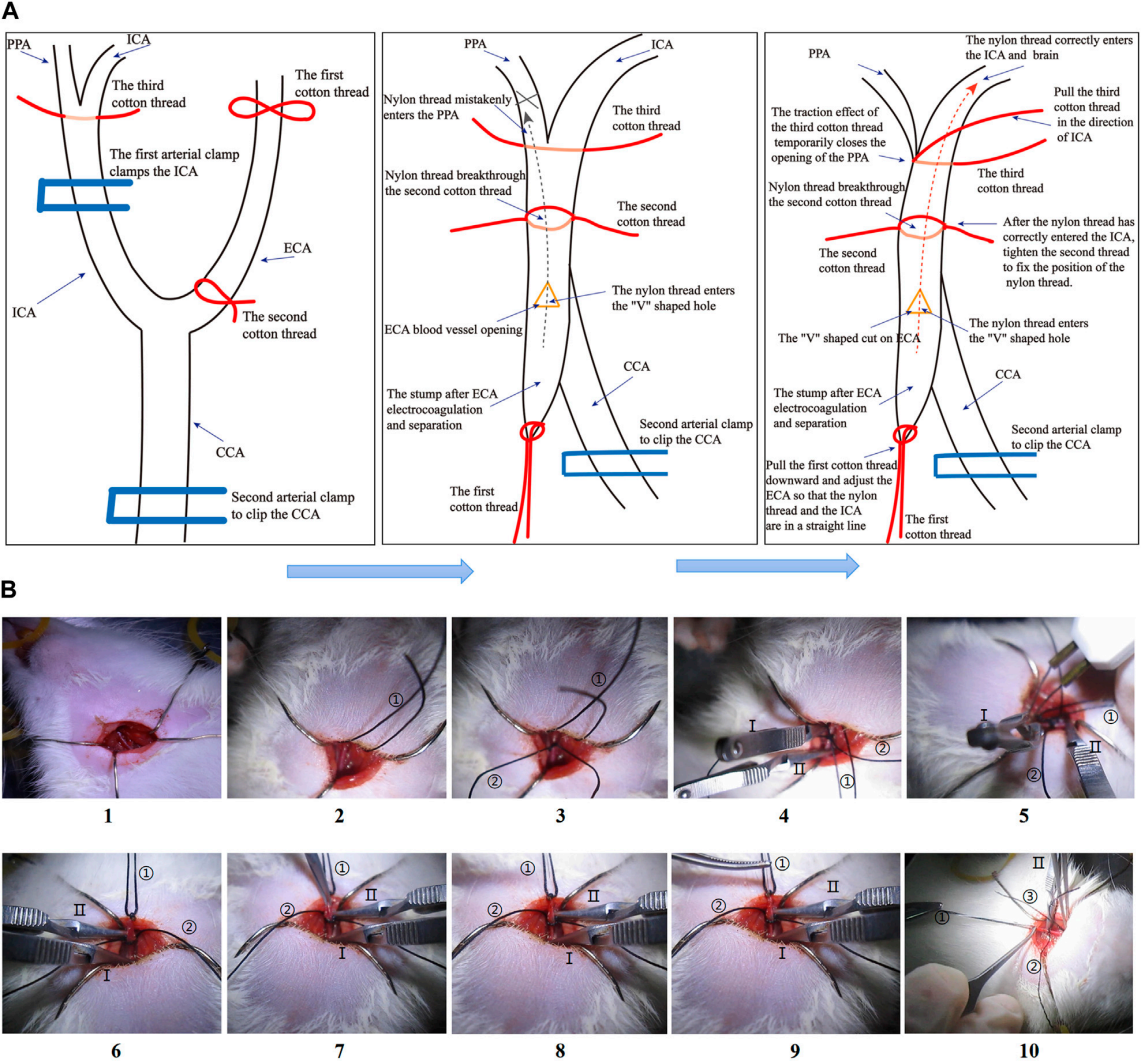
**(A)** Steps diagram of the Middle cerebral artery occlusion model (MCAO) in this study. **(A)** Schematic diagram of the MCAO operation steps. **(B)** The physical diagram of the operation steps of the MCAO. “①” in Figure 1B shows the first cotton thread, “②” shows the second cotton thread, and “③” shows the third cotton thread. “Ⅰ” in Figure 1B shows the first arterial clip, and “Ⅱ” shows the second arterial clip.

The models of focal CIRI were built with the MCAO method (Lyu et al., 2021; Huang et al., 2022). 4% isoflurane gives respiratory induction rats anesthesia, and 2% isoflurane maintains euthanasia. The left common carotid artery (CCA) was found and seen by dissection from surrounding tissues after skin cleansing and incision. The internal and external carotid arteries were then carefully exposed. After occluding the internal carotid artery with a microvascular clip and securing the distal end, the external carotid artery was transected 1 cm from the site of separation of the external carotid arteries (ECA) and internal carotid arteries (ICA). After removing the microvascular clip, a nylon monofilament (cat# 2636-A4, Beijing Cinontech, China) was introduced via the external carotid artery incision into the internal carotid arteries. Finally, when the monofilament was attached to the external carotid artery stump, resistance was felt. The monofilament was severed after 1.5 h of MCAO to accomplish reperfusion. In the current investigation, rats in the I/R and JDHXD-treated groups received blinded MCAO/R procedures, whereas rats in the sham group experienced simply the identical method without monofilament insertion. Compared with the previous method, we made detailed improvements in the surgical operation this time to shorten the operation time and improve the stability of the model (Figure 2). In addition, this modeling method is also helpful to avoid mistaken insertion of nylon monofilament into the pterygopalatine artery (PPA).

### Neurological function assessment

At 24 h and 72 h following brain infarction, the neurological deficit was measured by the Zea Longa tests. To rule out the effect of surgical failure, rats with MCAO/R but no obvious neurological abnormalities were eliminated. As previously indicated, Zea Longa tests were graded on a five-point scale (Longa et al., 1989). (0 = no deficiency, 1 = failure to extend right forepaw, 2 = right circling, 3 = right falling, 4 = No spontaneous walking with a low level of consciousness.) Before commencing the blinded testing, assessments were carried out without prior knowledge of the group allocation of the rats. Before the blinded testing, the assessments were done without knowing which group the rats were in.

### 2,3,5-Triphenyltetrazolium Chloride (TTC)

The brains were extracted, sliced into 3-mm-thick coronal sections, arranged on a mold, and then incubated at 37°C for 30 min in a solution comprising 2% TTC (cat# T8877-5G, Sigma, USA). Subsequently, digital photographs of the brain slices were taken (cat# HUAWEI Mate 30, HUAWEI, China), and the infarction percentage was calculated using ImageJ software (National Institutes of Health, USA). To ensure uniformity in the thickness of the slices, the brain was cut into five slices. Photography was conducted under laboratory fluorescent lighting, with no additional light sources or color processing. During the image-taking process, the same distance and lighting conditions were maintained as much as possible.

### HE staining

The histological evaluation of exfoliated rat brain tissue is conducted by hematoxylin-eosin (HE) staining. The initial step in this process is the immersion of the tissue in a 4% paraformaldehyde (PFA) solution for approximately 24 h. This is followed by the sectioning of the brain tissue for observation and subsequent dehydration through a graded ethanol series. After this procedure, the embedded brain tissue is then sliced at a thickness of 3 μm. Afterward, the tissue is subjected to dewaxing, which involves the removal of wax from the sections through a series of ethanol solutions. These slices were sequentially stained using hematoxylin and eosin (cat# G1003, Servicebio). The stained samples were examined using an upright optical microscope (NIKON ECLIPSE E100, Nikon, Japan) with an imaging system (NIKON DS-U3, Nikon, Japan).

### Nissl staining

Nissl staining was used to measure neuronal damage. Similar to the HE staining procedure, the tissue was initially placed in 4% PFA overnight, after which it was dehydrated with a graded alcohol series. It was then embedded in paraffin. The paraffin-embedded brain tissue was sectioned at a thickness of 3 μm. Subsequently, the tissue was incubated at 30°C for 5 minutes, during which it was stained with 1% toluidine blue (cat# G1032, Servicebio). After incubation in xylene for 10 min, sections were rinsed in a graded ethanol series and mounted. Finally, images of the same brain area were acquired using a light microscope and blinded analysis. The region surrounding the infarct core of the brain was selected as the site for quantifying the extent of neuronal damage. The corresponding hippocampal field, designated as CA-1, was identified for analysis. Efforts were made to maintain consistent section positions across different experimental groups.

### LC-MS analysis of JDHXD and Medicinal serum

In our current study, we collected the abdominal aorta blood of rats 30 min after administration of JDHXD and performed non-targeted LC-MS component detection to analyze the drug metabolites. Under a low-temperature environment, extract JDHXD and blank serum samples and JDHXD drug-containing serum samples. There were three groups of rat samples: the blank serum group, the JDHXD group, and the drug-containing serum group. The biological replicates of each group were 3 cases. The metabolite extraction method is as follows: the samples were thawed on ice, after 30 s vortex, the samples were centrifuged at 12000 rpm (RCF = 13800 (×g), R = 8.6 cm) for 15 min at 4 °C. 300 μL of supernatant was transferred to a fresh tube and 1,000 μL of extracted solution containing 10 μg/mL of internal standard was added, then the samples were sonicated for 5 min in ice-water. After placing 1 h in −40°C, the samples were centrifuged at 12000 rpm (RCF = 13800 (×g), R = 8.6 cm) for 15 min at 4°C. The supernatant was carefully filtered through a 0.22 μm microporous membrane, then 200 μL from each sample and pooled as QC samples. Store at −80°C until the UHPLC- MS analysis. 400 μL of plasma samples were added to 40 μL of hydrochloric acid (2 mol/L), then the mixture was vortexed for 1 min and followed by incubation for 15 min at 4°C. The vortex and incubated cycle were repeated for 4 times. Add 1.6 mL acetonitrile, then the mixture was vortexed for 5 min and the samples were centrifuged at 12000 rpm (RCF = 13800 (×g), R = 8.6 cm) for 5 min at 4°C. 1800 μL of supernatant was transferred to a fresh tube and nitrogen dried. The dried samples were reconstituted in 150 μL of 80% methyl alcohol containing 10 μg/mL of internal standard by vortex for 5 min. The constitution was then centrifuged at 12000 rpm (RCF = 13800 (×g), R = 8.6 cm) for 5 min at 4°C, and 120 μL of supernatant was transferred to a fresh glass vial for LC/MS analysis. LC-MS/MS analysis was performed on a UHPLC system (Vanquish, Thermo Fisher Scientific) with a Waters UPLC BEH C18 column (1.7 μm 2.1*100 mm). The sample injection volume was set at 5 μL. The flow rate was set at 0.5 mL/min. The mobile phase consisted of 0.1% formic acid in water (A) and 0.1% formic acid in acetonitrile (B). The multi-step linear elution gradient program was as follows: 0–11 min, 85%–25% A; 11–12 min, 25%–2% A; 12–14 min, 2%–2% A; 14–14.1 min, 2%–85% A; 14.1–15 min, 85%–85% A; 15–16 min, 85%–85% A. A Q Exactive Focus mass spectrometer coupled with Xcalibur software was employed to obtain the MS and MS/MS data based on the IDA acquisition mode. During each acquisition cycle, the mass range was from 100 to 1,500, the top three of every cycle were screened and the corresponding MS/MS data were further acquired. Sheath gas flow rate: 45 Arb, Aux gas flow rate: 15 Arb, Capillary temperature: 400°C, Full ms resolution: 70000, MS/MS resolution: 17500, Collision energy: 15/30/45 in NCE mode, Spray Voltage: 4.0 kV (positive) or −4.0 kV (negative).

### Western blot analysis

Based on the BCA protein assay kit (cat# ABW0104, Abiowell, China), the Western blot was used to measure and assess the amount of peri-infarcted protein levels. Briefly, proteins were transferred onto PVDF membranes (cat# IPVH00010, Millipore, USA). After the membranes were blocked with 4% nonfat milk for 2.5 h, the membranes were then incubated for 8 h at 4°C with multiple antibodies. Antibody information: NLRP3 (1:10,000, cat# ET1610-93, HUABIO), ASC (1:10,000, cat# 340097, ZENBIO), Caspase-1 (1:10,000, cat# ET1608-69, HUABIO), Gasdermin D (1:10,000, cat# HA721144, HUABIO), IL-18 (1:5,000, cat# 60070-1-Ig, Proteintech), IL-1β (1:5,000, cat# 511369, ZENBIO), IL-6 (1:5,000, cat# R1412-2, HUABIO), ULK1 (1:10,000, cat# 20986-1-AP, Proteintech), Beclin1 (1:10,000, cat#11306-1-AP, Proteintech), LC3 (Ⅰ/Ⅱ) (1:10,000, cat#12741, Cell Signaling), ZO-1 (1:10,000, cat# 21773-1-AP, Proteintech), P62 (1:5000, cat#HA721171, HUABIO), Beta Actin (1:10,000, cat# 20536-1-AP, Proteintech). The membranes were further incubated with horseradish peroxidase (HRP)-linked goat anti-rabbit IgG (1:10, 000, cat# E-AB-1003, Elabscience) or goat anti-mouse IgG secondary antibodies (1:10, 000, cat# E-AB-1001, Elabscience) for 1 h. After ECL photography, wash the membrane with stripping buffer for 20 min, and recycle the membrane. The blocking step should be conducted with 4% nonfat milk for 2.5 h, then incubated with another primary antibody. Repeat the previous steps to detect the expression level of another antibody. The amounts of proteins were detected and assessed using Bio-Rad Laboratories (BIO-RAD, USA). The Western blot images were analyzed by ImageJ software (NIH, USA).

### Immunohistochemical Staining

In brief, paraffin-embedded brain tissue was cut into 3 mm thick sections and dewaxed with xylene, then eluted with an ethanol gradient. The sections were placed in a microwave oven and antigen retrieval was conducted using sodium citrate. A blocking agent was then added, and the sections were incubated at 4°C for 30 min with 5% goat serum. The primary antibody, GSDMD (1:500, cat# 20770-1-AP, Proteintech), was applied, and the sections were incubated at 4°C overnight. The secondary antibody was applied at 37°C for 30 min. The DAB/DEC color development solution was applied for a period of five minutes, followed by a 3-minute incubation with hematoxylin staining solution. Hydrochloric acid-ethanol differentiation was conducted for 20 seconds, lithium carbonate solution was used for blueing, and the sections were then dehydrated and mounted. The sections were photographed under a microscope, and Image J software was employed to analyze the intensity of brown-yellow positive expression.

### Immunofluorescence

Briefly, the 3-mm-thick sections of brain tissues were blocked for 1 h with 5% goat serum. Thereafter, they were incubated for 24 h at 4°C with multiple primary antibodies. Antibody information: GSDMD (1:500, cat# 20770-1-AP, Proteintech), GSDMD (1:500, cat# 20770-1-AP, Servicebio), NLRP3 (1:200, cat# GB114320-100, Servicebio), caspase-1 (1:600, cat# GB11383-100, Servicebio), LC3 (Ⅰ/Ⅱ) (1:2000, cat# GB11124, Servicebio), NLRP3 (1:500, cat# ET1610-93, HUABIO), Caspase-1 (1:100, cat# 22915-1-AP, Proteintech), NeuN (1:1,000, cat# GB11138, Servicebio), DAPI (1:500, cat# G1012, Servicebio). DAPI glows blue by UV excitation wavelength 330–380 nm and emission wavelength 420 nm; FITC glows green by excitation wavelength 465–495 nm and emission wavelength 515–555 nm; CY3 glows red by excitation wavelength 510–560 nm and emission wavelength 590 nm. The figures were taken by fluorescence microscope (Nikon, Japan).

### Analytical statistics

Continuous data were presented as mean ± standard deviation (SD) using the GraphPad Prism software, and one-way ANOVA was conducted to ascertain significant differences between groups (GraphPad Prism Software Inc., San Diego, CA, USA). A significance level of *p* < 0.05 was employed to determine statistical significance. Data that significantly deviate from the mean will be eliminated. Outliers are identified through the Grubbs test, and the number of repeated tests is increased for values that deviate from the norm or for different research personnel to verify the results. If a significant deviation is confirmed, outliers are removed.

## Results

### Metabolites analysis of JDHXD medicated serum

The Supplementary Material showed the untargeted LC-MS positive/negative ion spectrum of JDHXD blood metabolites (Supplementary Material S1). Because LC-MS yields total anion and total cation maps, there is masking of the wave peaks of different substances and does not facilitate direct labeling of the metabolite peak spectra directly in the ion maps, the specific comparison of the blood-entry metabolites is shown in Table 2. Table 2 illustrates the comparison of the metabolites. Table 2 showed 30 possible metabolites with a similarity score of >0.98 in the JDHXD medicated serum component spectrum analyzed by LC-MS. Then based on the ten plant combinations of JDHXD: *Pueraria edulis* Pamp [Fabaceae], *Paeonia veitchii* Lynch [Paeoniaceae], *Prunus persica (L.)* Batsch [Rosaceae], *Anethum graveolens* L. [Apiaceae], *Rehmannia glutinosa (Gaertn.)* DC. [Orobanchaceae], *Carthamus tinctorius* L. [Asteraceae], *Forsythia suspensa (Thunb.)* Vahl [Oleaceae], *Bupleurum falcatum* L. [Apiaceae], *Citrus medica* L. [Rutaceae], *Glycyrrhiza glabra* L. [Fabaceae]). A total of 1,174 possible metabolites derived from the JDHXD-containing serum were selected (Composite Score >0.6). The closer the spectrum similarity score is to 1, the higher the structural similarity. The Table 2 shows the metabolites’ name, original plant source, and metabolite spectrum composite score. In addition, the table also showed the assay values for metabolites with a composite score greater than 0.98 in blank serum, JDHXD drug solution, and JDHXD-containing serum. It is important to explain that since non-targeted LC-MS will detect a large number of possible substances, only possible substances with a similarity of >0.98 are shown in Table 2. This is why some theoretically important active substances are not shown in Table 2.

**TABLE 2:** LC-MS/MS analysis of metabolites in JDHXD medicated serum (Composite Score >0.98).

No.	Name	Botanical drugs	Compo-site score	Blank serum 1	Blank serum 2	Blank serum 3	JDHXD1	JDHXD2	JDHXD3	JDHXD serum 1	JDHXD serum 2	JDHXD serum 3
**1**	2-Octenoic acid	*Bupleurum falcatum* L. [Apiaceae]	1	33526386.99	31918941.05	30366077.07	27679886.45	26047574.2	24637554.42	27034216.46	26327116.06	38194633.09
**2**	5-HYDROXYMETHYL-2-FURANCARBOXYLIC ACID	*Rehmannia glutinosa (Gaertn.)* DC. [Orobanchaceae]	1	2295488.679	1450542.933	0	47674890.75	46527312.41	45174820.9	0	5855370.021	1755579.779
**3**	Baicalein	*Carthamus tinctorius* L. [Asteraceae]; *Paeonia veitchii Lynch* [Paeoniaceae]	1	137603888.2	127435055.9	112581544.4	172283.2092	212336.696	156548.459	54268574.01	47023934.24	10016018.54
**4**	Indole-3-carboxylic acid	*Glycyrrhiza glabra* L. [Fabaceae]	1	13469142.89	13203008.53	1,3942008.28	938468.7319	948294.1136	1071481.947	10427139.83	10232425.35	3633702.402
**5**	Methylnissolin-3-O-glucoside	*Anethum graveolens* L. [Apiaceae]	1	0	0	0	24772478.77	23077088.44	22967588.59	0	116848.4373	0
**6**	p-Hydroxybenzaldehyde	*Pueraria edulis* Pamp [Fabaceae]	1	12749816.97	14434648.55	10935133.36	245007095.3	247008477	248652406	9160790.757	12444179.78	9300150.971
**7**	Quercetin	*Pueraria edulis* Pamp [Fabaceae] *Bupleurum falcatum* L. [Apiaceae]; *Carthamus tinctorius* L. [Asteraceae]	1	0	103208.5123	44908.55502	24938053.79	25916371.08	24771563.58	155264.0965	41445.76569	572401.8785
**8**	Scutellarein	*Carthamus tinctorius* L. [Asteraceae]	1	24613.20225	44530.70708	36508.43751	39425330.14	44126473.96	42195209.75	895059.6164	1193062.241	1479907.622
**9**	Genistein	*Pueraria edulis* Pamp [Fabaceae]	0.999999	521618.1842	734657.2362	1015771.452	12448055.45	12604829.35	12234904.15	692576.0342	909797.7863	283964.5742
**10**	Pyrocatechol	*Carthamus tinctorius* L. [Asteraceae]; *Prunus persica* (L.) Batsch [Rosaceae]	0.999979769	1792675.296	1384688.008	1105512.573	7269450.303	7389564.14	4310365.589	420742.8586	569956.6155	669592.9478
**11**	Adenine	*Anethum graveolens* L. [Apiaceae]	0.999903462	45080650.33	26234116.14	36324013.37	437433834.8	440172444.8	450436588.3	7527368.949	2353952.37	11944398.68
**12**	Liquiritigenin	*Pueraria edulis* Pamp [Fabaceae]; *Glycyrrhiza glabra* L. [Fabaceae]	0.997646692	336233.2093	1520972.604	1332806.392	400249.906	347373.701	604859.6631	33012207.55	29884405.33	4889048.021
**13**	Daidzein	*Pueraria edulis* Pamp [Fabaceae]	0.997220308	2505838.863	2775918.922	2929516.206	1879687960	1892177727	1903152190	8916035.238	9355704.749	2074290.392
**14**	Xanthotoxol	*Citrus medica* L. [Rutaceae]; *Anethum graveolens* L. [Apiaceae]	0.995398154	36926.79674	46667.67757	67998.31834	7631357.438	7869663.346	8017132.314	33288.52626	23352.52818	0
**15**	Isorhamnetin	*Glycyrrhiza glabra* L. [Fabaceae]; *Bupleurum falcatum* L. [Apiaceae]	0.995101077	0	0	0	16959827.81	16268316.34	17705896.29	718125.0212	786451.8819	930092.657
**16**	Scopoletin	*Forsythia suspensa (Thunb.)* Vahl [Oleaceae]; *Anethum graveolens* L. [Apiaceae]; *Glycyrrhiza glabra* L. [Fabaceae]; *Bupleurum falcatum* L. [Apiaceae]	0.994920231	3523516.481	8497248.766	10094242.82	6800668.766	13988634.03	5619104.177	9739483.531	9207170.189	7637459.356
**17**	(+/−)-Jasmonic acid	*Citrus medica* L. [Rutaceae]	0.993650846	1753070.721	2265081.225	2082550.086	426540.3759	367005.5974	468690.1548	1348686.564	1554043.158	2145586.288
**18**	Biochanin A	*Pueraria edulis* Pamp [Fabaceae]	0.993143385	440662.2446	160976.2658	146674.3868	100822826.1	94854103.23	97635226.94	973690.2118	1295050.31	716833.6495
**19**	Methyl hexadecanoate	*Pueraria edulis* Pamp [Fabaceae]; *Paeonia veitchii* Lynch [Paeoniaceae]; *Prunus persica* (L.) Batsch [Rosaceae]	0.992738	7880680.801	8535370.148	8955699.694	34947.47829	11954.25219	31124.7922	13847800.96	13810169.31	4548178.284
**20**	Wogonin	*Forsythia suspensa* (Thunb.) Vahl [Oleaceae]	0.992622154	46158333.28	46449517.26	51646546.05	158776245.8	162275820.2	161536285.4	376697167.7	376936701	5645137.914
**21**	7-Hydroxycoumarin	*Citrus medica* L. [Rutaceae]; *Anethum graveolens* L. [Apiaceae]; *Forsythia suspensa* (Thunb.) Vahl [Oleaceae]	0.992348385	1985240.076	1736999.464	1855661.178	396904414.6	419959144.6	423509537.4	1192292.781	1957527.618	2247232.647
**22**	Genkwanin	*Glycyrrhiza glabra* L. [Fabaceae]	0.991977154	2606722.886	2530362.482	2049485.537	68686232.5	69161039.5	67201769.7	26985912.45	24348705.02	5769563.191
**23**	magnolol	*Citrus medica* L. [Rutaceae]	0.991036923	116667.3412	54730.46163	27195.76422	12225491.42	10810012.82	11666127.15	145676.0231	388400.8612	149898.6976
**24**	Kaempferide	*Carthamus tinctorius* L. [Asteraceae]	0.989199846	495096.6106	492660.8302	404650.9711	912147.3486	1114992.206	1188308.663	876900.6075	684449.6833	176742.502
**25**	alpha-Linolenic acid	*Bupleurum falcatum* L. [Apiaceae]; *Carthamus tinctorius* L. [Asteraceae]; *Prunus persica* (L.) Batsch [Rosaceae]	0.983700692	11457160.96	16259517.7	18252916.84	2128424.436	2185558.256	2164212.649	15802196.7	13628633.97	18885641.98
**26**	Nicotinic acid	*Anethum graveolens* L. [Apiaceae]	0.983485385	8732229.227	8962208.648	10506670.64	13565913.01	13326116.13	13092406.35	8996147.805	8554744.259	10736957.42
**27**	2-Ethylphenol	*Anethum graveolens* L. [Apiaceae]	0.982149	3018656.272	2265248.912	2895775.329	7723605.129	8139428.666	7543579.113	3220141.108	2092897.664	3386825.874
**28**	eriocitrin	*Citrus medica* L. [Rutaceae]	0.980994231	0	0	0	2727903.033	3145598.286	2642769.072	0	0	0
**29**	Choline [M+]	*Anethum graveolens* L. [Apiaceae]; *Forsythia suspensa* (Thunb.) Vahl [Oleaceae]	0.980813	837414910.2	974847415.3	952590406.2	2371366284	2309712551	2441160317	973100819.5	870448804.8	972883048.3
**30**	Palmatine	*Pueraria edulis* Pamp [Fabaceae]; *Glycyrrhiza glabra* L. [Fabaceae]	0.980601769	55410.34584	59111.41605	145233.4135	240442449.3	226089832.4	230532979.3	116481.8016	209755.6923	248578.5025

### JDHXD mitigated neurological impairments and cerebral infarction

**FIGURE 3:**
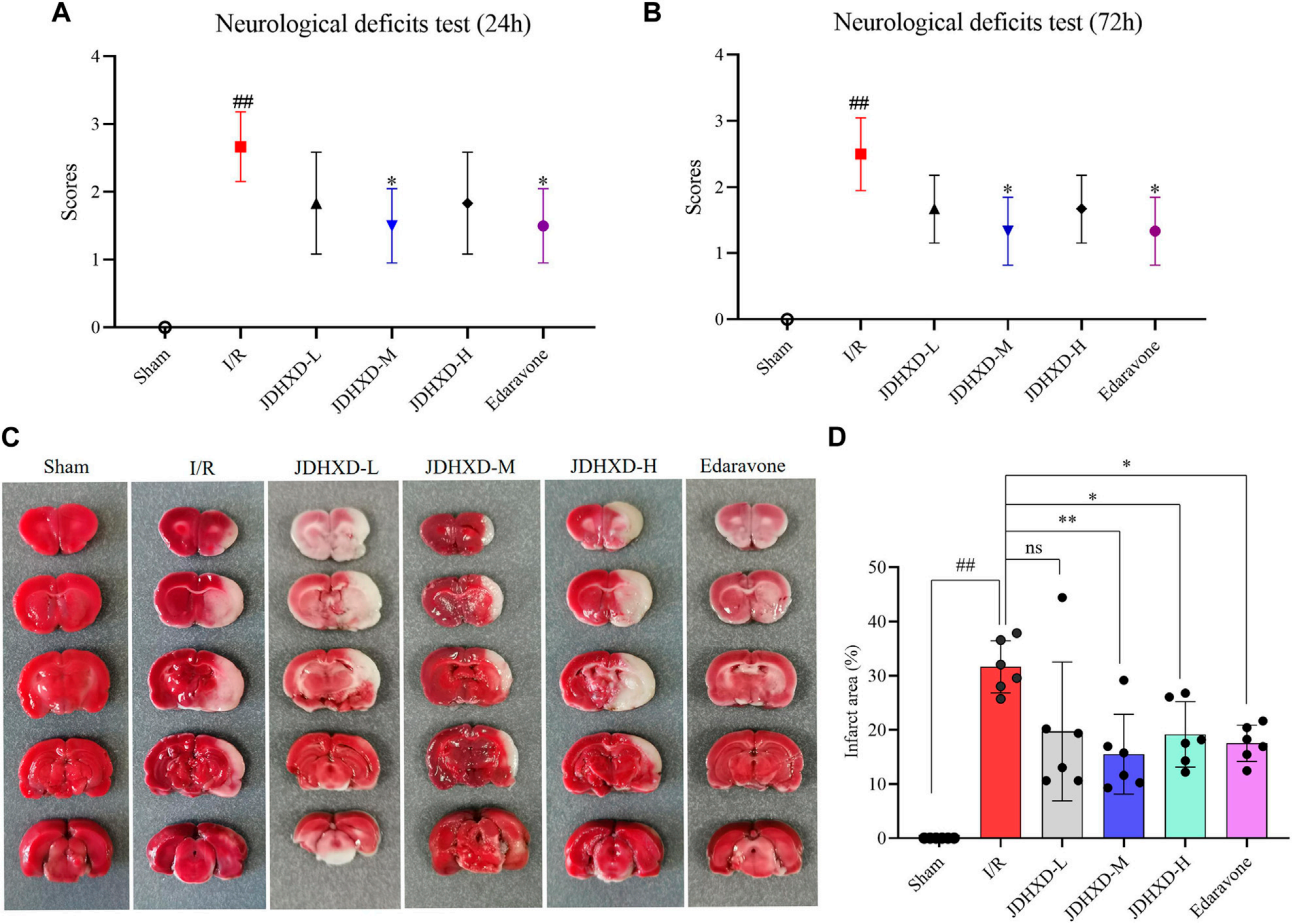
JDHXD reduced neurological deficits and cerebral infarction following CIRI. **(A)** Test for neurological impairments 24 h after reperfusion. **(B)** Test for neurological impairments 72 h after reperfusion. Both **(A)** and **(B)** demonstrated that a modest dosage of JDHXD decreased neurological deficits in rats 24 and 72 h after reperfusion, n = 6. **(C)** Typical pictures of TTC staining of rat brain tissue in each group. Non-infarcted tissue is stained red by the TTC solution, while the infarcted area is white. **(D)** Comparison of the brain infarct area to different groups, n = 6. ^##^ *p* < 0.01 vs. Sham group; ^**^ *p* < 0.01 vs. I/R group. ^*^ *p* < 0.05 vs. I/R group. (JDHXD, Jie-Du-Huo-Xue decoction; CIRI, cerebral ischemia-reperfusion injury; TTC, 2,3,5-Triphenyltetrazolium Chloride).

Compared with the I/R group, the JDHXD-M treated group showed significantly lower neurological deficit scores at 24 and 72 h after reperfusion (*p* < 0.05). The group administered the positive drug exhibited a similar trend to that observed in the JDHXD-M group. In the current experiment, we optimized the MCAO modeling method, shortened the operation time, and made the model more stable. Figure 3 illustrated that rats in the sham group displayed no cerebral infarction, whereas those in the I/R group showed evident cerebral ischemia (*p* < 0.01). JDHXD-M group revealed significantly less cerebral infarct area than the I/R group (*p* < 0.01). Different from the previous results, the JDHXD-H group also exhibited significant statistical differences (*p* < 0.05). Based on the findings from the initial stage of this study, the subsequent tests selected the suitable dose of JDHXD (JDHXD-M) to assess its potential effects on the interplay between neuroinflammation-related pyroptosis and autophagy.

### JDHXD reduced neuronal damage after CIRI in rats

**FIGURE 4:**
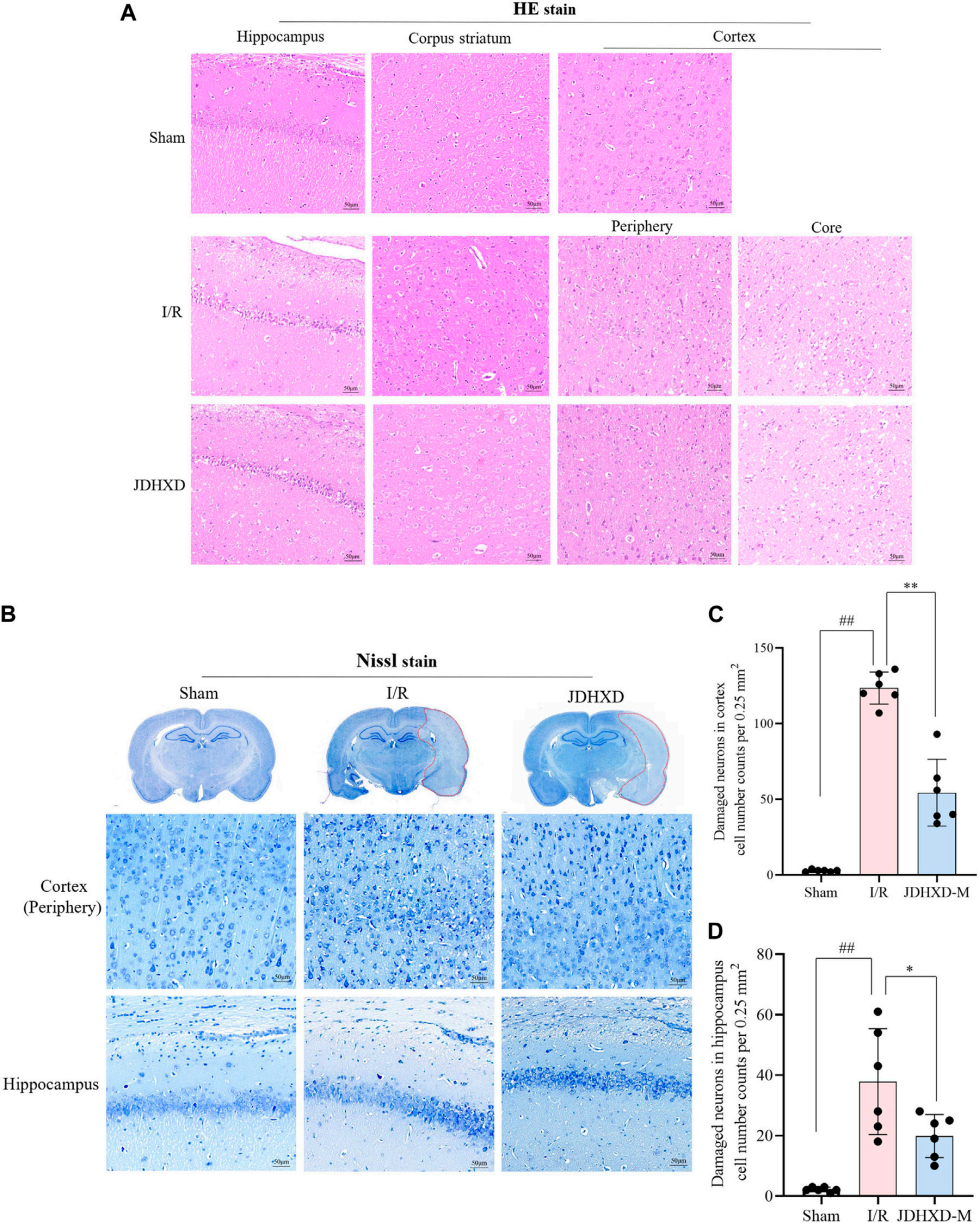
JDHXD ameliorated neuronal injury 72 h post-CIRI in rats. **(A)** Representative images of HE staining in the ischemic cortex, hippocampus, and corpus striatum. scale bars = 50 μm, n = 3. **(B)** Panoramic scan with Nissl staining and partial map of cortex and hippocampus-CA1. **(C, D)** Quantitative assessment of neuronal damage in the cortex and hippocampus, n = 6. ^*##*^ *p* < 0.01 vs. Sham group; ^*^ *p* < 0.05 vs. I/R group.. (The edge of the infarct core was selected as the observation field for the purpose of counting the number of damaged neurons. This was because, in the infarct core, there was no significant difference in the number of neurons with abnormal morphology between the I/R group and the JDHXD group. The presence of damaged neurons can be identified by the following characteristics: cell shrinkage, reduced synapses, obvious cell morphology collapse, disordered cell arrangement, and widened tissue gaps around cells under Nissl staining.)

The above results demonstrated that JDHXD could reduce neurological impairment and cerebral infarction induced by CIRI. To further demonstrate the neuroprotective effect of JDHXD, we evaluated the morphological changes of neurons in the central-infarct zone, peri-infarct zone, hippocampus CA-1, and corpus striatum (Figures 4A, B). Compared with the sham group, the cells in the infarct core area were arranged disorderly in the I/R group. Typical cell shrinkage and nuclear pyknosis occur. Vacuoles surrounding cells indicate the presence of tissue edema. As illustrated in Figures 4C, D, JDHXD treatment reduced the number of morphologically abnormal cells in the cortical penumbra compared with the I/R group (*p* < 0.01). Furthermore, JDHXD treatment reduced morphologically abnormal cell numbers in the hippocampal CA-1 compared with the I/R group (*p* < 0.05). However, the protective effect of JDHXD was not reflected in the infarct center. There was no significant difference in the infarct core between the I/R and JDHXD groups (*p* > 0.05). These results are consistent with the indicative significance of neurological function assessment and cerebral infarct area measurement.

### JDHXD reduces the neuronal pyroptosis in the penumbra 3 Days following CI

**FIGURE 5:**
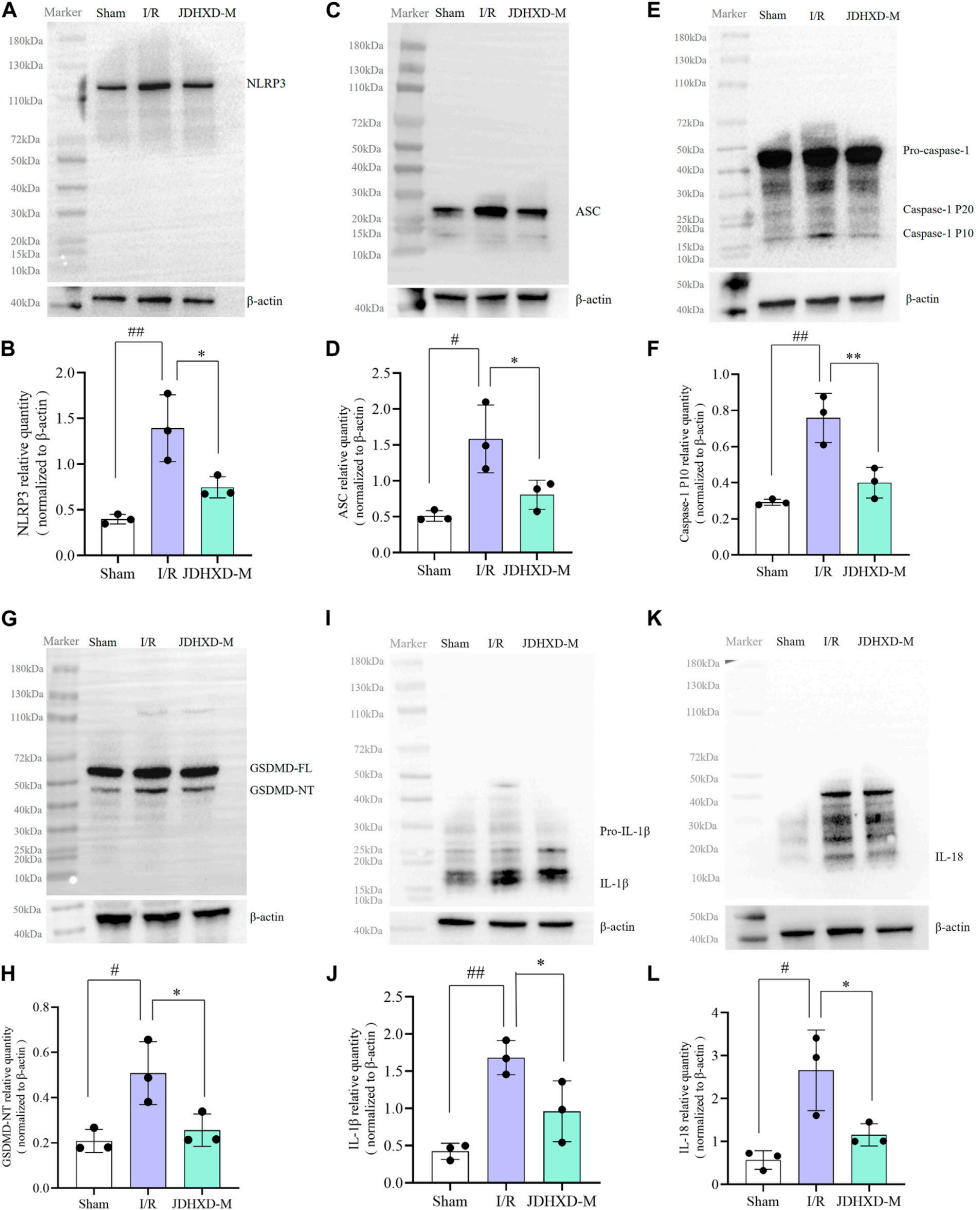
Expression of pyroptosis-related molecules in the peri-infarct region following JDHXD-M therapy. **(A, B)** Western blot of the NLRP3, ASC, pro-caspase-1, caspase-1 P10, GSDMD-FL, GSDMD-NT, IL-1β, IL-18 and β-actin proteins in peri-infarct tissues. **(C–L)** Statistical chart of relative expression of NLRP3, ASC, Caspase-1 P10, GSDMD-NT, IL-1β, IL-18. Data are expressed as means ± SD, n = 3, ^*##*^ *p* < 0.01 vs. Sham group; ^*#*^ *p* < 0.05 vs. Sham group; ^**^ *p* < 0.01 vs. I/R group; ^*^ *p* < 0.05 vs. I/R group. (GSDMD, gasdermin D; GSDMD-FL, Gasdermin D full length; GSDMD-NT, Gasdermin D N terminal; IL, interleukin; NLRP3, nucleotide-binding oligomerization domain-like receptor pyrin domain containing 3; ASC, apoptosis-associated speck-like protein containing a card; SD, standard deviation).

To better represent the expression of pyroptosis-related proteins, we showed the Western blot image of the entire PVDF membrane. As shown in Figures 5A–D, JDHXD-M inhibited the expression of NLRP3 and ASC around the infarct on the third day after CI in rats (*p* < 0.05). This indicates that JDHXD inhibits the expression of inflammasomes that induce pyroptosis. Consistent with previous findings, JDHXD-M markedly reduced the expression levels of caspase-1 P10 (*p* < 0.01), and GSDMD-NT (*p* < 0.05) (Figures 5E–G). GSDMD-NT can oligomerize to form holes on the cell membrane and is the direct executor of pyroptosis. Caspase-1 P10 is one of the effector fragments after caspase-1 cleavage, which is upstream of GSDMD and can cleave GSDMD-FL into GSDMD-NT. This indicates that JDHXD-M inhibits the classical pathway pyroptosis around the infarct on the third day after CI in rats. Similar results have been observed with downstream signaling molecules of pyroptosis, including IL-18 and IL-1β (Figure 5I–L). Compared with the IR group, the expression of IL-18 and IL-1β in the JDHXD-M group was significantly reduced (*p* < 0.05). IL-1β and IL-18 are downstream inflammatory factors released by pyroptotic GSDMD pores, causing inflammatory damage to brain tissue.

**FIGURE 6:**
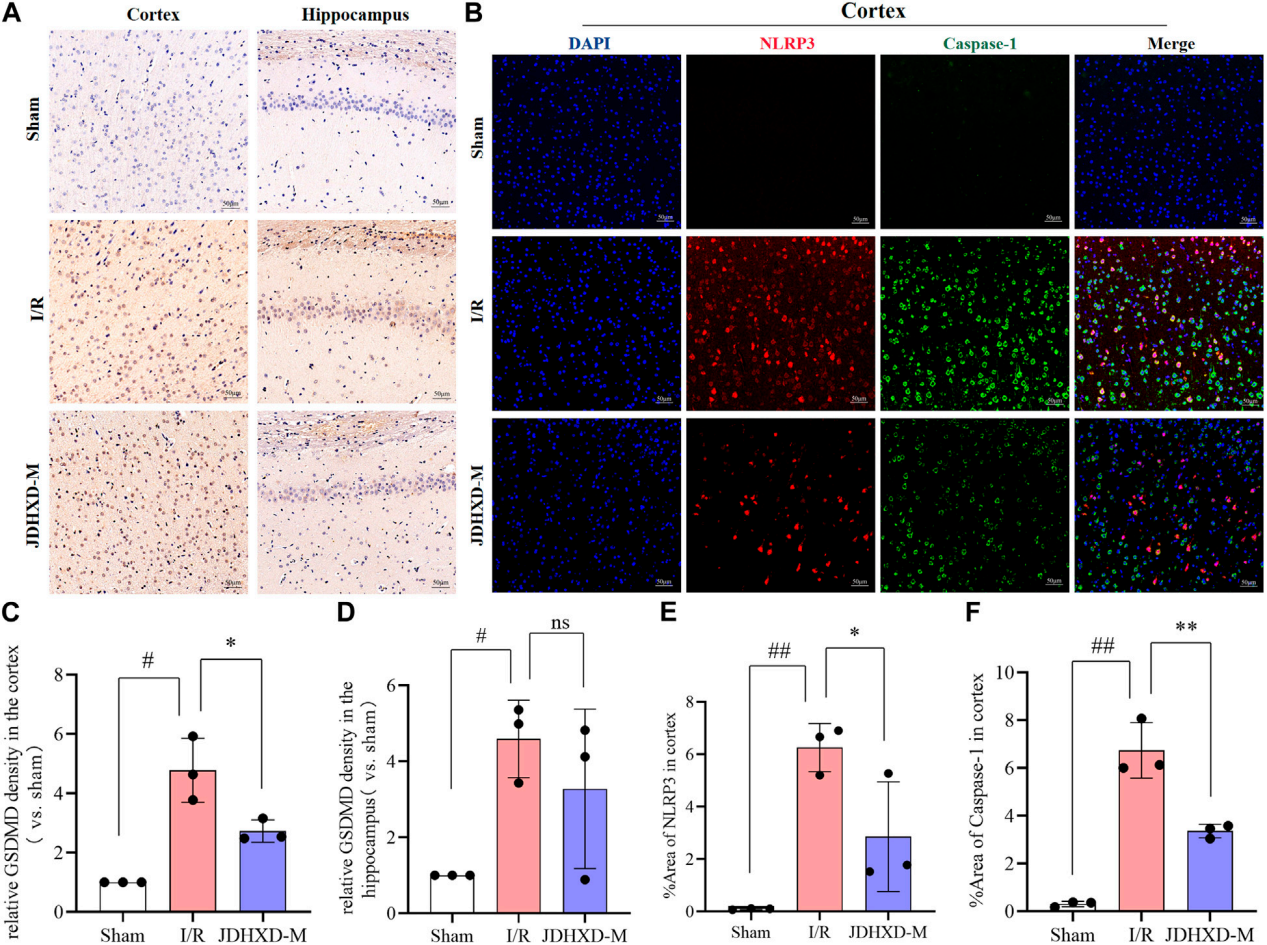
Immunohistochemistry and immunofluorescence results of relative expression of pyroptosis-related proteins. **(A)** Immunohistochemistry results of GSDMD in the penumbra on the third day after CI in rats. Brown represents the positive expression of GSDMD, and blue represents the nucleus. **(B)** Immunofluorescence results of NLRP3 and caspase-1 in the penumbra zone on the third day after CI in rats, NLRP3 (red), caspase-1 (green), DAPI (blue). **(C)** Relative expression of GSDMD in the penumbra of ischemic cortex in immunohistochemistry experiment. **(D)** Relative expression of GSDMD in the ischemic hippocampus in immunohistochemistry experiment. **(E)** Expression area % of NLRP3 in ischemic cortical penumbra in immunofluorescence experiment. **(F)** Expression area % of caspase-1 in the ischemic cortical penumbra in immunofluorescence experiment. Data are expressed as means ± SD, n = 3, ^*##*^ *p* < 0.01 vs. Sham group; ^*#*^ *p* < 0.05 vs. Sham group; ^**^ *p* < 0.01 vs. I/R group; ^*^ *p* < 0.05 vs. I/R group. (GSDMD, gasdermin D; NLRP3, nucleotide-binding oligomerization domain-like receptor pyrin domain containing 3; DAPI, 4′,6-diamidino-2-phenylindole).

We further examined the localization of pyroptosis markers such as NLRP3, caspase-1, and GSDMD by immunofluorescence and immunohistochemistry. The immunohistochemistry showed that brown-yellow GSDMD positive expression can be observed around the infarction in the ischemic hemisphere (Figure 6A). We discovered that the expression of NLRP3 and caspase-1 increased in the infarcted hemisphere in I/R and JDHXD-M groups (Figure 6B). Three days after CI in rats, NLRP3 could be observed in the infarct core, while the expression of caspase-1 and GSDMD was mainly in the peri-infarct penumbra. These findings align with our prior investigations and those of other research teams. Compared with the I/R group, the relative density of GSDMD in the ischemic hemisphere of the JDHXD-M group was significantly reduced. (Figure 6C, D, *p* < 0.05). Compared with the IR group, the fluorescence area percentage of NLRP3 and caspase-1 in the ischemic hemisphere of the JDHXD-M group was significantly reduced (Figure 6E, F, *p* < 0.05). In summary, JDHXD inhibits the expression of pyroptosis.

### Loss of pyroptotic GSDMD protein May contribute to adverse outcomes of CIRI

**FIGURE 7:**
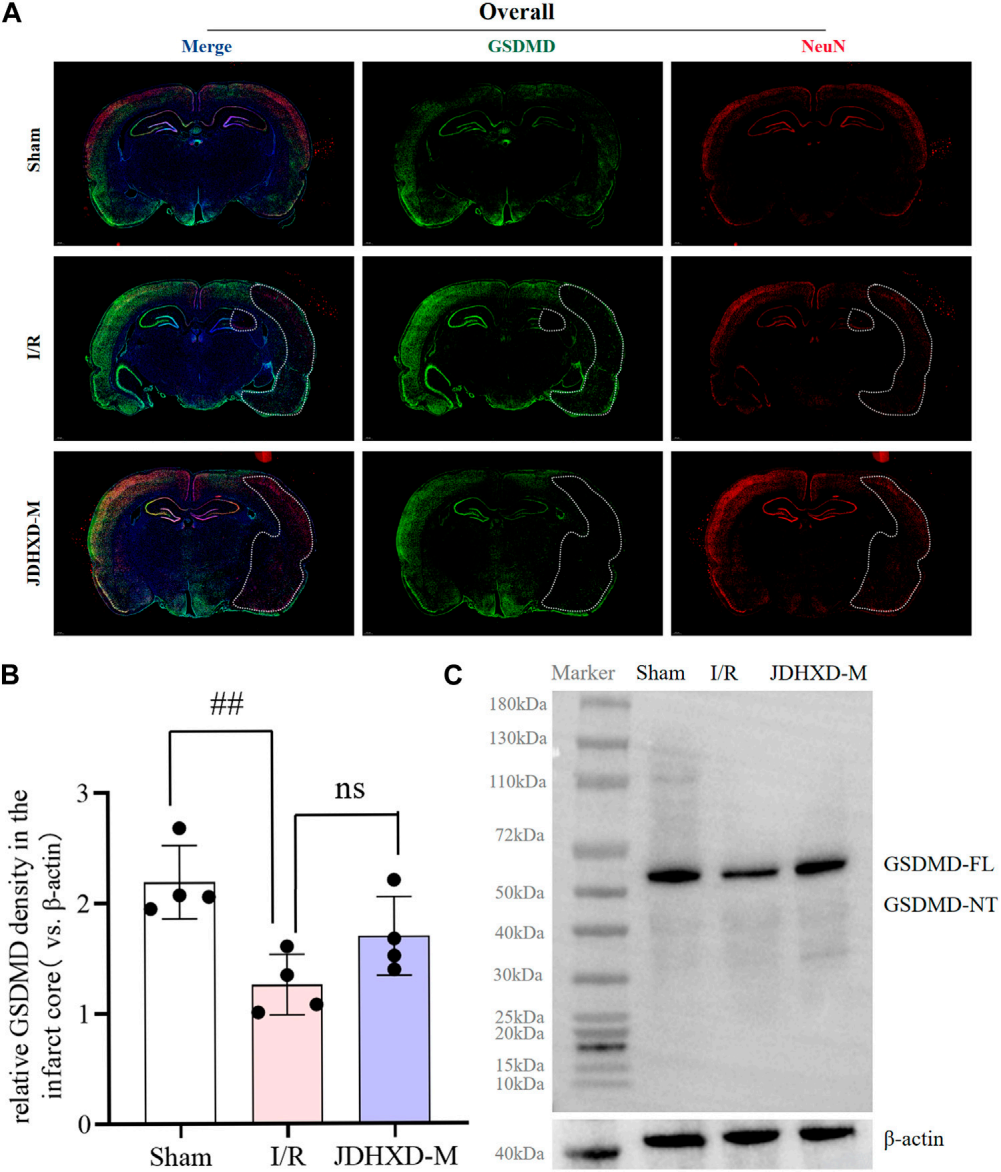
The key pyroptosis execution protein GSDMD is lost in the infarct core area on the third day after CI in rats. **(A)** Immunofluorescence panoramic scan of GSDMD and NeuN. GSDMD (green), NeuN (red), DAPI (blue). The white dotted line is the core area of infarction. **(B)** Relative expression of GSDMD protein in the infarct core. n = 4, scale bar = 50 μm ^#^ *p* < 0.05 vs. Sham group; ^*^ *p* < 0.05 vs. I/R group. **(C)** Typical Western blot image of GSDMD protein in the infarct core area. (GSDMD, gasdermin D; DAPI, 4′,6-diamidino-2-phenylindole; JDHXD, Jie-Du-Huo-Xue decoction; CIRI, cerebral ischemia-reperfusion injury; NeuN, neuron-specific nuclear protein).

During performing GSDMD fluorescence staining, we observed an intriguing phenomenon that, to the best of our knowledge, has not been previously reported. In this preliminary report, we present our observations on this phenomenon. Our findings indicate that the green GSDMD fluorescence is distributed symmetrically in the left and right cerebral hemispheres. In contrast, the green GSDMD fluorescence is absent in the infarct core of the I/R group and JDHXD-M group (Figure 7A). It is crucial to highlight that previous studies have described this phenomenon as a high expression of GSDMD in the penumbra. However, the results of our panoramic scan demonstrate that the expression intensity of GSDMD fluorescence in the non-infarcted hemisphere and penumbra is nearly identical, and is significantly higher than that observed in the infarct core area. To ensure the accuracy and reproducibility of the results, we replaced the GSDMD antibodies with different species antibodies and performed the procedures by different personnel. The results remained consistent with those previously described. Furthermore, western blot analysis of brain tissue from the core of the infarction revealed a significant reduction in GSDMD-FL expression in the I/R group (*p* < 0.05). However, the protein bands of GSDMD-NT in the infarct core of the sham operation group, I/R group, and JDHXD group were not visible (Figures 7B, C). The results of Western blotting were aligned with those of double immunofluorescence, thereby providing a basis for proposing a phenomenon that was inconsistent with previous findings. This may indicate that the loss of pyroptotic GSDMD protein after CI may also contribute to the adverse outcomes of CI. It is possible that excessive activation of GSDMD around the infarct and massive loss of the infarct core may result in damage after CI. Although further investigation is required to elucidate the underlying mechanism, we have elected to present this valuable finding for consideration.

### JDHXD inhibited the expression of NLRP3 protein of pyroptosis not by promoting autophagy

**FIGURE 8:**
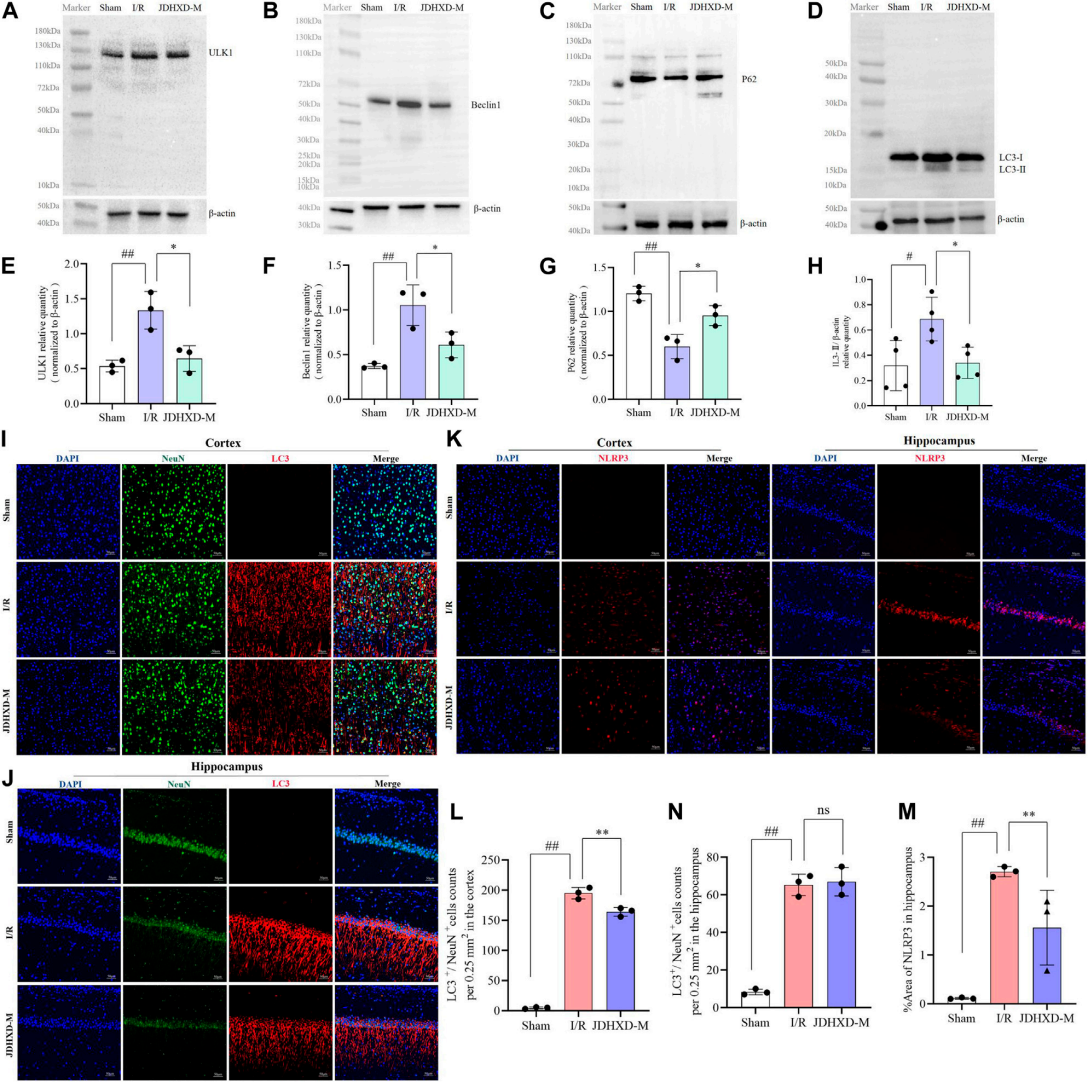
Expression levels of autophagy-related molecules in the peri-infarct region following treatment with JDHXD-M. **(A)** Western blot of the ULK-1. **(B)** Western blot of beclin1. **(C)** Western blot of P62. **(D)** Western blot of LC3-Ⅰ/Ⅱ. **(E–H)** Relative expression of ULK1, beclin1, P62, LC3-Ⅰ/Ⅱ in the infarct core. Data are expressed as means ± SD, n = 3, scale bar = 50 μm ^##^ *p* < 0.01 vs. Sham group; ^**^ *p* < 0.01 vs. I/R group, ^*^ *p* < 0.05 vs. I/R group. **(I)** Double immunofluorescence staining of the cortex with NeuN (green) and LC3 (red) on day 3 after MCAO. **(J)** Double immunofluorescence staining of the hippocampus with NeuN (green) and LC3 (red) on day 3 after MCAO. **(L)** The histogram quantified the LC3^+^/NeuN^+^ cells of the cortex. **(M)** The histogram quantified the LC3^+^/NeuN^+^ cells of the hippocampus. **(K)** Immunofluorescence results of NLRP3 in the penumbra on the third day after CIRI in rats, NLRP3 (red) and DAPI (blue). **(N)** Expression area % of NLRP3 in immunofluorescence experiment. Data are expressed as means ± SD, n = 3, scale bar = 50 μm ^*##*^ *p* < 0.01 vs. Sham group; ^**^ *p* < 0.01 vs. I/R group; ^ns^ *p* > 0.05. (LC3, lipidation of microtubule-associated light chain 3; ULK1, unc-51-like autophagy activating kinase 1; NLRP3, nucleotide-binding oligomerization domain-like receptor pyrin domain containing 3; CIRI, cerebral ischemia-reperfusion injury; NeuN, neuron-specific nuclear protein; JDHXD-M, Jie-Du-Huo-Xue decoction-medium dose).

Similar to pyroptosis, autophagy can also regulate cellular inflammatory responses through the inflammasome (Wang et al., 2020). Autophagy is a double-edged sword. Moderate autophagy during CI is conducive to maintaining cell function under the low energy supply state of ischemia and hypoxia (Wei et al., 2012). Insufficient autophagy is not conducive to the clearance of inflammatory products and stress in the early stage of ischemia, but excessive autophagy can cause tissue damage (Wang et al., 2018). The state of autophagy in CI is both an enemy and a friend, so there is no conclusion on whether autophagy is good or bad in CI(Nabavi et al., 2019; Orellana-Urzúa et al., 2020). Major studies suggested that autophagy is enhanced after CI, and appropriate inhibition of autophagy can reduce ischemia-reperfusion injury. Our study also supported the above view. To explore the intervention effect of JDHXD on neuronal autophagy during the initial phase of CIRI, we assessed the expression of autophagy-related proteins. Our findings revealed that on the third day following CI in rats, the level of autophagy was higher than the normal level. Compared to the sham group, the expression of the autophagy-initiating factor ULK1 was found to be significantly elevated in the IR group (*p* < 0.01). Furthermore, the expression of beclin1 demonstrated a significant upregulation following CI (*p* < 0.01). Similarly, the level of LC3-II, a marker of autophagy, was elevated in the ischemic hemisphere (*p* < 0.05). JDHXD downregulated the ULK1, beclin1, and LC3-Ⅱ in rats with CIRI, which indicates that JDHXD inhibited autophagy in the ischemic hemisphere (Figures 8A-H, *p* < 0.05).

We used immunofluorescence to label the autophagy protein LC3 and NeuN to observe the level of neuronal autophagy, and the results supported the above conclusions (Figure 8I, L, *p* < 0.01). Interestingly, the expression of LC3 in the hippocampus was not different between the IR and the JDHXD-M groups (Figure 8J, N, *p* > 0.05). At the same time, the expression level of NLRP3 did not increase due to the decrease in autophagy levels (Figure 8K, M, *p* < 0.01). The above results suggest that JDHXD suppressed the excessively increased autophagy level after CI.

## Discussion

Timely and reliable national stroke surveillance remains a significant challenge (Ziaeian et al., 2022). Due to the strict window period of treatment and the inpatient system to be improved, acute ischemic stroke continues to be the primary cause of mortality and disability (Mosconi and Paciaroni, 2022; Ziaeian et al., 2022). The pathological mechanism of CI is very complex, involving multiple disease stages such as the early and recovery stages. Different cell death modes such as apoptosis, autophagy, and pyroptosis crosstalk each other (Zhao S. et al., 2021). Oxidative stress, immunity, inflammation, thrombosis, and many other links are also involved (Yingze et al., 2022; Maïer et al., 2023; Wang et al., 2023). Above all, developing drugs to treat CI became much harder. New research confirms that inflammatory response is the main thread during all stages of stroke (Maïer et al., 2023). Emerging studies have shown that traditional medicine plays an important role in attenuating the inflammatory damage caused by CI, which undoubtedly suggests the importance of further searching for ethnic medicine alternative therapies for CI.

**FIGURE 9:**
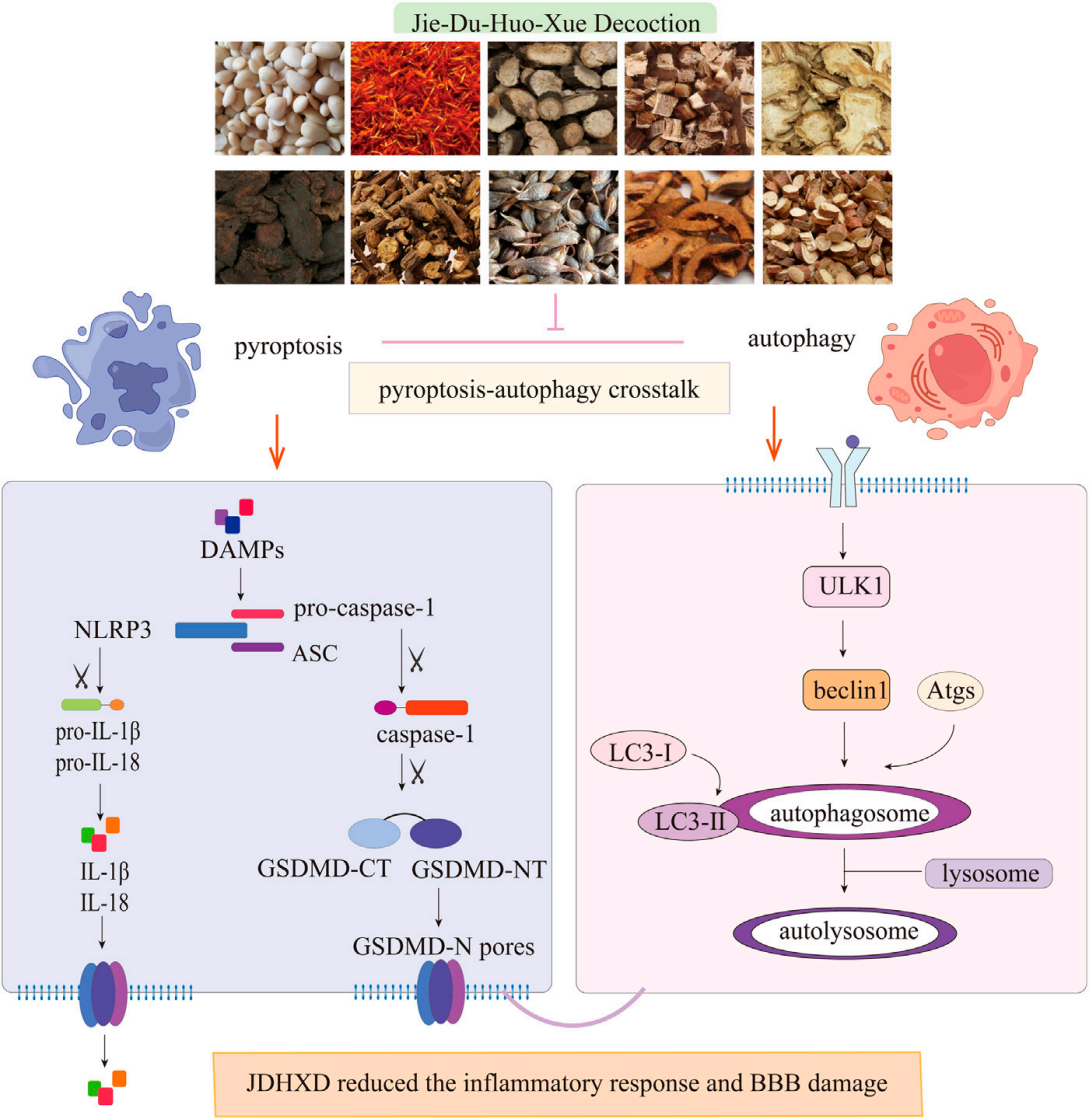
JDHXD against CIRI in rats. JDHXD reduced pyroptosis and autophagy. JDHXD consists of 10 botanical drugs (*Pueraria edulis* Pamp [Fabaceae], *Paeonia veitchii* Lynch [Paeoniaceae], *Prunus persica* (L.) Batsch [Rosaceae], *Anethum graveolens* L. [Apiaceae], *Rehmannia glutinosa (Gaertn.)* DC. [Orobanchaceae], *Carthamus tinctorius* L. [Asteraceae], *Forsythia suspensa (Thunb.)* Vahl [Oleaceae], *Bupleurum falcatum* L. [Apiaceae], *Citrus medica* L. [Rutaceae], *Glycyrrhiza glabra* L. [Fabaceae]). Our results showed that JDHXD inhibited both focal death and autophagy. On the one hand, JDHXD inhibited the classical pyroptosis pathway, suppressed the expression of NLRP3/caspase-1/GSDMD, and reduced the production of IL-1β and IL-18. On the other hand, JDHXD also inhibited the expression of ULK1/beclin1/LC3-II on the autophagy pathway. NLRP3 serves as a nexus linking focal death and autophagy, and JDHXD inhibition of NLRP3 does not work by promoting autophagy. (LC3, lipidation of microtubule-associated light chain 3; ULK1, unc-51-like autophagy activating kinase 1; NLRP3, nucleotide-binding oligomerization domain-like receptor pyrin domain containing 3; GSDMD-CT, gasdermin D C terminal; GSDMD-NT, Gasdermin D N terminal; IL, interleukin).

In this work, we observed the regulatory effect of JDXHD on programmed death in the acute phase of CI based on the previous confirmation that JDHXD inhibits microglial pyroptosis in the acute phase of CI. Before studying the mechanism of JDHXD’s protective effect on CI, we detected the blood metabolites of JDHXD by LC-MS. We detected and analyzed 30 possible metabolites (Composite Score > 0.98). Previous studies have given some evidence that JDHXD metabolites inhibit programmed cell death. For example, Pueraria lobata root can dilate cerebral blood vessels, enhance cerebral blood flow, ameliorate cerebral circulation, and stabilize cerebral vascular function, thereby improving blood and oxygen supply to brain tissue and cells, and reducing the occurrence of neurological dysfunction (Hongyun et al., 2017). Chinese thorowax root may enhance depression-like behavior and decrease hippocampus neuron death following CI, probably by boosting the expression of BDNF, p-CREB, and Bcl-2 while reducing the levels of Bax and Caspase-3 (Wang A. R. et al., 2021). Licorice can promote cell proliferation, migration, and angiogenesis, and reduce mitochondrial membrane potential damage and apoptosis. Simultaneously, liquiritigenin can decrease the expression of associated adhesion molecules and mitigate the generation of reactive oxygen species. Mechanistically, liquiritigenin can activate the Keap1/Nrf2 antioxidant pathway, thereby preserving the integrity of the BBB (Li et al., 2021). Ferulic acid, for example, has been shown to encourage the development of new blood vessels *in vivo*. Ferulic acid boosted the expression of VEGF and platelet-derived growth factor (PDGF) (Lin et al., 2010). Rehmannia glutinosa can improve the post-ischemic repair signaling pathway (Fu et al., 2022). This investigation demonstrated that JDHXD exerts an inhibitory effect on neuronal pyroptosis and autophagy following CI in rats. JDHXD reduces the expression of NLRP3, caspase-1 P10, GSDMD-NT, IL-1β, and IL-18. JDHXD also inhibited the expression of autophagy-related factors, such as ULK1, beclin1, and LC3-II on the third day after CI (Figure 9).

Pyroptosis and autophagy are both forms of programmed cell death (Adhami et al., 2007; Xue et al., 2019). Among them, pyroptosis is a pro-inflammatory cell death mode. The traditional view is that autophagy has nothing to do with inflammatory response, but the latest research shows that autophagy is also involved in the regulation of inflammatory response (Su et al., 2016). The conventional and the non-canonical pathways are the two primary pathways of pyroptosis. In CI, pyroptosis is primarily the conventional pathway (Yu et al., 2021). Pyroptosis signals in the canonical pathways activate several cytoplasmic sensor proteins and encourage the formation of various inflammasomes (Gou et al., 2021). CI causes a strong neuroinflammatory response in a strict temporal and spatial order (Gelderblom et al., 2009). During the first 24 h of CIRI, the inflammation is mainly confined to the intravascular compartment (Stoll et al., 2022). This may be driven by local platelet and leukocyte-derived DAMPs and chemokines (Kollikowski et al., 2022). During pyroptosis, the NLRP3 inflammasome is activated by DAMPs produced in CIRI (Chang et al., 2020). And then NLRP3 cleaves pro-caspase-1 into activated caspase-1. Activated caspase-1 promotes the maturation of IL-18 and IL-1β and activates GSDMD. GSDMD is one of the gasdermin protein family (McKenzie et al., 2018; Van Opdenbosch and Lamkanfi, 2019). It has an autoinhibitory domain. When caspase-1 is cleaved, the active N-terminal is exposed, and the N-terminal gathers on the cell membrane to form a ring-shaped membrane hole, causing the cell to collapse (Burdette et al., 2021; Wang J. et al., 2021). Small inflammatory molecules such as IL-18 and IL-1β can be released from the pores of GSDMD, and then recruit more pro-inflammatory factors to gather, induce an inflammatory cascade reaction, and aggravate cerebral ischemic injury (Schneider et al., 2017; Evavold et al., 2018; Luan et al., 2020). Our study proved that JDHXD can inhibit many links of pyroptosis in the classical pathway in the rat MCAO model. It can not only inhibit NLRP3 but also inhibit the active structure of caspase-1 P10 and GSDMD-N terminal, as well as the downstream inflammatory factors of pyroptosis. Inhibition of neuronal pyroptosis by JDHXD can help relieve neuroinflammation in the acute phase of CIRI, protect the brain parenchyma, and reduce neurological impairment and cognitive impairment after CIRI. JDHXD inhibits pyroptosis of the NLRP3/caspase-1/GSDMD pathway, which is of great value in inhibiting CI. Because studies have shown that preventive inhibition of NLRP3 inflammasome can maintain its early therapeutic effect into the subacute phase, inhibition of NLRP3 inflammasome in the acute phase of CI directly affects inflammation in the ischemic penumbra after stroke (Bellut et al., 2023). Early treatment effects tend to have long-term immunomodulatory effects, ultimately leading to better neurological outcomes. In addition, inhibition of NLRP3 can reduce secondary infarct growth in small and medium-sized CI (Bellut et al., 2023). Therefore, JDHXD may have a delayed immunomodulatory effect by inhibiting NLRP3 during the acute phase of CI, but further studies are needed. In addition, JDHXD has an important protective effect on the prognosis of stroke by inhibiting IL-1β and IL-18 produced downstream of pyroptosis. IL-18 has been shown in multiple clinical studies to be a potent proinflammatory cytokine with atherogenic properties (Goldstein et al., 2006). In addition, higher plasma IL-1β levels are associated with the most severe damage in patients with ischemic stroke (Tirandi et al., 2023). JDHXD’s inhibition of IL-1β and IL-18 may reduce the diffusion of interleukins through the damaged BBB.

Interestingly, our study proposes a hypothesis that is different from previous studies. The loss of the pyroptosis execution protein GSDMD protein in the ischemic core area may lead to adverse consequences of CI. Although many previous studies have shown that GSDMD is lowly expressed in the core of cerebral ischemic infarction and highly expressed in the periphery of the infarction (Luo et al., 2022), to our knowledge, we are the first to show that GSDMD is also highly expressed in the healthy brain contralateral to the ischemic brain. Our panoramic scanning results show that the expression intensity of GSDMD fluorescence in the non-infarcted hemisphere and penumbra is almost the same, and is much higher than that in the infarct core area. Different GSDMD antibodies were used and staining operations were performed by different personnel, and the same results were obtained. In addition, Western blot results also support the low expression of GSDMD-FL in the core area of infarction. We are very excited about this finding, which is contrary to previous findings. At the same time, our previous results on microglial pyroptosis also support the enhanced expression of GSDMD in the infarct penumbra. We speculate that pyroptosis GSDMD may have a double-edged sword effect like autophagy. Just like “Yin” and “Yang” in philosophy are opposite and unified with each other. Excessive activation of GSDMD at the infarct edge will aggravate post-ischemic brain injury, and the loss of GSDMD protein in the core area of infarction after cerebral ischemia may also lead to adverse outcomes of CIRI. We do not rule out the possibility that the following bias may occur: the fluorescent antibody cannot label GSDMD-NT very accurately but instead displays GSDMD-FL. To eliminate the interference of the above factors, we used antibodies of different species produced by two different formulas in immunofluorescence. If our hypothesis that GSDMD is lost in the infarct core is confirmed in subsequent studies, it may provide new clues to the role of pyroptosis in CI. Pyroptosis may not be entirely a pro-inflammatory injury during CIRI, and GSDMD may also have a potential protective mechanism against CI. Although we have not yet studied the mechanism of this phenomenon in depth, we chose to report this valuable finding.

In comparison to pro-inflammatory pyroptosis, the regulation of inflammation by autophagy and its role in CI is more confusing. There has been debate over whether autophagy has a protective or harmful effect (Plaza-Zabala et al., 2017). By far the most prevalent description is that autophagy is a double-edged sword (Wei et al., 2012). Both positive and negative autophagy regulation can improve neuroprotection *in vivo* models of CI (Wang et al., 2022; Zheng et al., 2022; Zhai et al., 2023). Autophagy is a self-protective catabolic mechanism in cells. Some misfolded proteins and damaged organelles are destroyed and recycled via this mechanism to preserve cellular equilibrium (Adhami et al., 2007). The activation or inhibition of autophagy under ischemia and hypoxia is still controversial, and there is no shortage of contrary research results. Autophagy is commonly thought to be triggered in reaction to nutrient deprivation or metabolic stress, aiming to sustain tissue equilibrium by recycling dysfunctional proteins and organelles (Zhao J. et al., 2021; Ji et al., 2022). Briefly, autolysosomes arise as a result of the sequestration of cytoplasmic material into autophagosomes and fusion with lysosomes. Under nutritional shortage, AMPK is activated, inactivating mTORC1 or phosphorylating different serine residues of ULK1 (Zhai et al., 2023). However, some studies have also proved that autophagy is inhibited under nutrient deprivation (Zheng et al., 2022). Our research results indicate that neuronal autophagy is triggered by CI and it can be inhibited by JDHXD. JDHXD suppressed the expression of the autophagy promoter ULK1, as well as beclin1 and LC3-II. In our current study, on day 3 after CI in rats, autophagy in neurons in the cerebral ischemic penumbra was higher than normal, suggesting that ischemia and hypoxia lead to excessive autophagy, which causes excessive neuronal death. JDHXD-M intervention can promote neuronal autophagy and reduce neuroinflammation.

Updated research changes the old view that autophagy is not involved in inflammatory responses. Emerging research suggests that autophagy may protect against inflammation (Wang et al., 2020). The mechanisms by which autophagy inactivates the inflammasome are diverse, including clearance of ASC and IL-1β, and selective targeting of ROS to lysosomes for clearance by mitophagy (Li et al., 2015; Shibutani et al., 2015). However, our findings do not support the above view because autophagy promoted the inflammatory damage of CI in our study. The concurrent activation of LC3-II and beclin-1 proteins, along with NLRP3, IL-1β, and caspase-1, is functionally implicated in the progression and pathogenesis of CIRI, characterized by concurrent activation of autophagy, pyroptosis, and inflammation. These three processes are often triggered in a sequential and interconnected order. Both pyroptosis and autophagy are active in the cortical area of the ischemic penumbra. On the one hand, NLRP3 is involved in the initiation of pyroptosis, and on the other hand, NLRP3 is activated by the acute and subacute injury signals after CI in rats, and NLRP3 subsequently cleaves caspase-1 and GSDMD to induce pyroptosis. At this time, the level of autophagy was activated due to ischemia and hypoxia, and the activated autophagy did not inhibit the phosphorylation of NLRP3 and ASC. This promotes the inflammatory response following CI. The intervention of JDHXD suppressed the expression of autophagy markers ULK1, Beclin1, and LC3-II. On the third day after CI, the activation of autophagy did not reduce NLRP3, and activated NLRP3 could activate pro-inflammatory programmed cell death.

Our results support the widely accepted view that autophagy is overactivated after CI and that inhibition of NLRP3 can suppress the expression of pyroptosis and autophagy (Liu et al., 2021). Interestingly, some studies have suggested that enhancing mitochondrial autophagy and reducing NLRP3 inflammasome activation has a neuroprotective effect on rats after CI (Ji et al., 2022). In addition, some reports promoting autophagy can inhibit pyroptosis in myocardial ischemia (Zhang et al., 2023). The inconsistency of pathophysiological mechanisms of the same cell death pattern in different tissues has considerable research prospects. It is postulated that this phenomenon may be attributed to the specific effects of NLRP3 in diverse tissues, degrees of ischemia, and cell types. The clearance ability of autophagy on NLRP3 may be different in different tissues, organs, and cells, thus affecting the interaction between autophagy and pyroptosis. Although JDHXD did not activate autophagy and inhibit pyroptosis as we speculated, it inhibited pyroptosis and autophagy at the same time, indicating that JDHXD inhibits pyroptosis possibly by directly inhibiting the cleavage of NLRP3 and GSDMD-NT.

Our study confirmed that JDHXD inhibits pyroptosis and autophagy and alleviates inflammatory damage. However, there are some deficiencies and limitations in the experimental design. First, in the western blot experiment, the housekeeping protein we chose was β-actin. New studies suggested that in neurological diseases, housekeeping proteins such as β-actin and GAPDH may be changed after injury or in neuropathological states (Li and Shen, 2013). Our current experiments have not verified that the expression of β-actin is constant in all samples of the CI model, so the possible experimental bias caused by β-actin cannot be ruled out. Secondly, due to the limitations of experimental conditions, our experiments could not directly observe the pores formed by GSDMD oligomerization but only observed the expression of GSDMD by western blot and immunofluorescence. Future research can improve the experimental design from the above perspectives.

## Conclusion

In conclusion, our study demonstrated that JDHXD inhibited the expression of pyroptosis and autophagy on the third day of CI. Furthermore, JDHXD exhibited an anti-CI effect. Our findings also indicated that the inhibitory effect of JDHXD on pyroptosis was not mediated by the promotion of autophagy. In a surprising twist, our findings revealed that GSDMD expression was low in the infarct core but high in the peri-infarct and healthy brain regions, a result that challenges previous assumptions.

## Data Availability

The raw data supporting the conclusions of this article will be made available by the authors, without undue reservation.

## Abbreviations

CI, cerebral ischemia; CIRI, cerebral ischemia-reperfusion injury; SMCs, smooth muscle cells; CNS, central nervous system; JDHXD, Jie-Du-Huo-Xue decoction; DAMPs, damage-associated molecular patterns; LC3, lipidation of microtubule-associated light chain 3; ASC, apoptosis-associated speck-like protein containing a card; BBB, blood-brain barrier; ROS, reactive oxygen species; EC, endothelial cell; ECM, extracellular matrix; GSDMD, gasdermin D; ULK1, unc-51-like autophagy activating kinase 1; NLRP3, nucleotide-binding oligomerization domain-like receptor pyrin domain containing 3.
