# Supplementary material for: Gasdermin D could be lost in the brain parenchyma infarct core and a pyroptosis-autophagy inhibition effect of Jie-Du-Huo-Xue decoction after stroke
Source: Front Pharmacol. 2024 Jul 29;15:1449452. doi: 10.3389/fphar.2024.1449452 (PMC11320715; doi:10.3389/fphar.2024.1449452)
Supplement: Supplementary file 1 [file DataSheet1.docx]

| 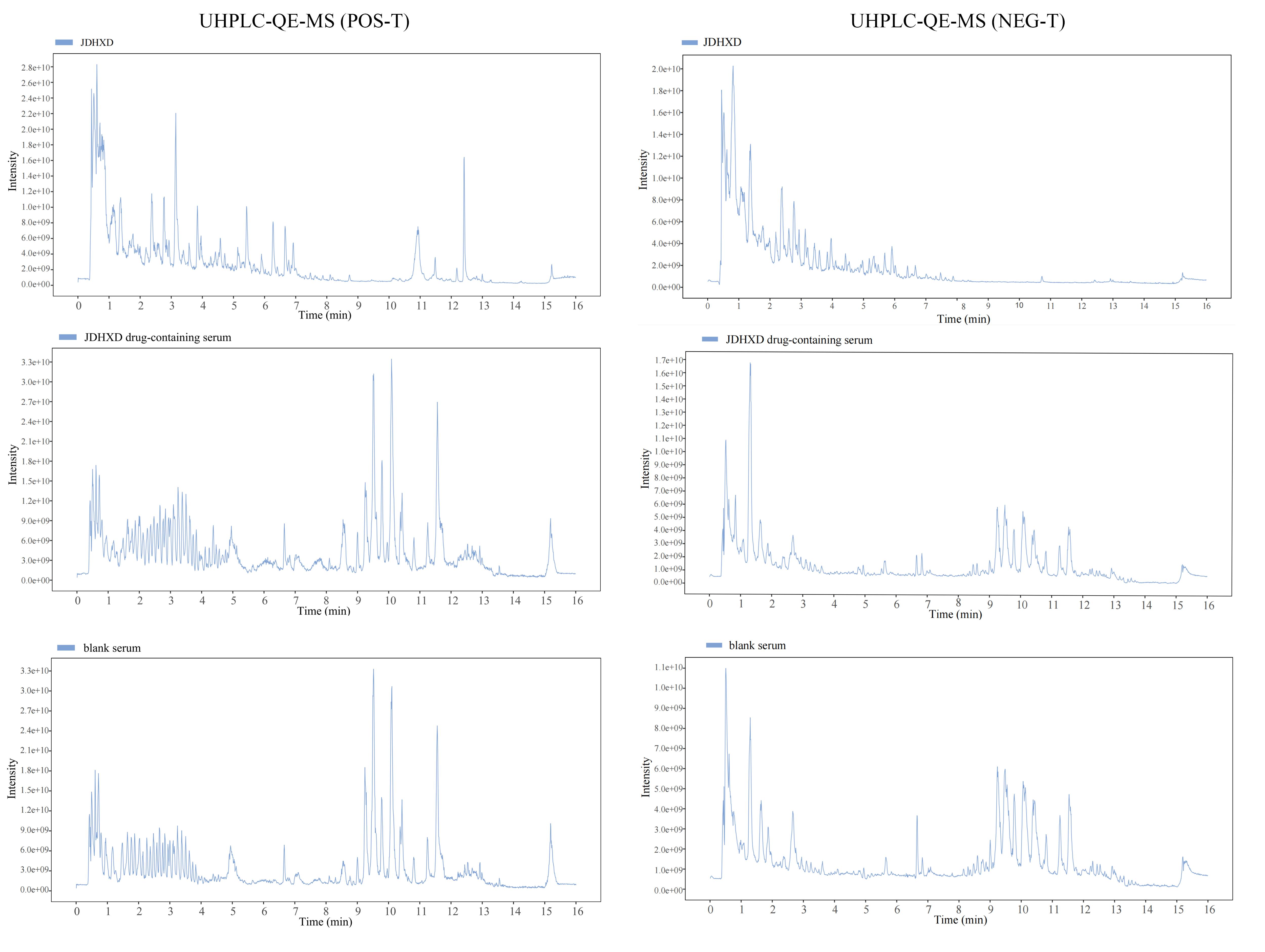 |
| --- |
| **Supplementary file\| Analyses of JDHXD’s primary chemical metabolites.** Chromatogram of JDHXD by the LC-MS positive/negative ion spectrum of JDHXD and blood serum.  Because LC-MS produces a total anion map and a total cation map, different substance peaks may be obscured. The specific metabolites entering the blood that were compared are shown in Table 2.  (JDHXD, Jie-Du-Huo-Xue decoction; LC-MS, Liquid Chromatography-Mass Spectrometry) |
